# The Use of Generative Artificial Intelligence (AI) in Academic Research: A Review of the Consensus App

**DOI:** 10.7759/cureus.87297

**Published:** 2025-07-04

**Authors:** Olukayode E Apata, Oi-Man Kwok, Yuan-Hsuan Lee

**Affiliations:** 1 Educational Psychology, Texas A&M University, College Station, USA; 2 Department of Learning Technologies, University of North Texas, Denton, USA

**Keywords:** academic integrity, consensus app, ethical concerns, reporting practices, transparency

## Abstract

Consensus App is an academic search engine designed to change how researchers access and synthesize information. It helps researchers quickly browse the growing body of academic literature by offering insights at both the topic and paper levels. We evaluate the Consensus App's potential to transform academic research, its ethical implications, and the reasons behind its underrepresentation in academic literature. We seek to provide a balanced perspective on the app’s current and future influence in academic research. This paper is based on a rapid review of the literature to see how the Consensus App is used and reported in the literature. Our review followed the Preferred Reporting Items for Systematic Reviews and Meta-Analyses (PRISMA) guidelines. We focused on identifying applications, benefits, and ethical concerns related to the Consensus App. The search was conducted on December 23, 2024, across 210 academic databases. The databases from which articles were retrieved include Web of Science (N=6), MEDLINE (N=2), Academic Search Ultimate (N=1), and Fuente Académica Plus (N=1). In addition to the database searches, five additional editorials were identified through targeted manual searches of high-impact journals. In total, 10 papers were included in the final review. ChatGPT-4.5 was used to assist in synthesizing key themes across the articles, focusing on application, benefits, and ethical concerns related to the Consensus App and the broader use of artificial intelligence (AI) in scholarly work. The reviewed articles revealed that the use of the Consensus App is surprisingly low, which may suggest underreporting by its users. Researchers may also not be aware of it. These studies showed how the app has been limitedly used in the literature. Despite its advantages, we identified ethical concerns in the reviewed studies. Despite its potential, the Consensus App remains underutilized and significantly underreported in academic literature. Therefore, it is important for academic institutions, journal editors, and researchers to collaboratively develop standardized reporting guidelines when AI is involved in the process of manuscript development. The eventual goal is to lead to a more transparent reporting of AI usage in research.

## Introduction and background

For decades, evidence synthesis has relied on manual, keyword-based searches in databases such as PubMed and Scopus, a time-consuming process that often misses semantically relevant studies and slows the uptake of new knowledge. To overcome these limitations, a new generation of artificial intelligence (AI) tools has emerged to accelerate and enrich literature retrieval. The rapid rise of generative AI technologies such as ChatGPT, which launched in late 2022, has quickly transformed many aspects of daily life, including education, communication, and scholarly research. Unlike other generative AI apps, Consensus App is an academically oriented search engine specifically designed to change how researchers access and synthesize information [1]. It has mainly targeted the needs of researchers in the academic environment. A report [2] stated that the app uses advanced large language models (neural networks trained on massive text corpora to understand and generate human-like language) and purpose-built vector search technology (a retrieval method that matches queries to documents based on semantic similarity rather than exact keyword overlap) to search the most relevant academic papers. It helps researchers quickly browse the growing body of academic literature by offering insights at both the topic and paper levels. The Consensus App is an academic AI tool designed to retrieve and synthesize peer-reviewed literature. While its developers outline several features intended to support researchers, such features were not discussed or reported in the studies included in this review. However, despite the platform’s rapid uptake, peer-reviewed evaluations of the Consensus app remain unavailable. No empirical study has yet examined whether its promised advantages translate into measurable improvements in search quality, researcher workflow, or ethical soundness. Addressing this gap is critical for guiding responsible adoption and informing future tool development.

Given the growing number of AI-powered tools available to support academic research, it is necessary to conduct a review of the Consensus App to evaluate its unique capabilities and limitations. While other tools, such as OpenAI’s DeepResearch, provide generative AI outputs for summarizing or explaining content, the Consensus App is distinct in that it specifically draws from peer-reviewed academic articles and conference proceedings and presents scientifically grounded claims with supporting evidence, which enhances transparency and relevance in literature synthesis. These capabilities make the app especially suitable for evidence-based academic work, in contrast to broader AI systems that usually hallucinate and may not prioritize scientific rigor or database-backed evidence. Therefore, evaluating the Consensus App is essential for understanding how it contributes to trustworthy research synthesis in comparison to other generative AI platforms such as ChatGPT, Gemini, and Meta-AI.

In light of the documented lack of peer-reviewed evidence, this review presents a balanced discussion of the benefits and drawbacks of the Consensus App, addressing its ability to improve research efficiency and the ethical concerns associated with its use. We also provide recommendations for researchers, developers, and academic institutions to maximize the app’s potential while addressing its limitations.

## Review

Methodology

Overview

**Figure 1:**
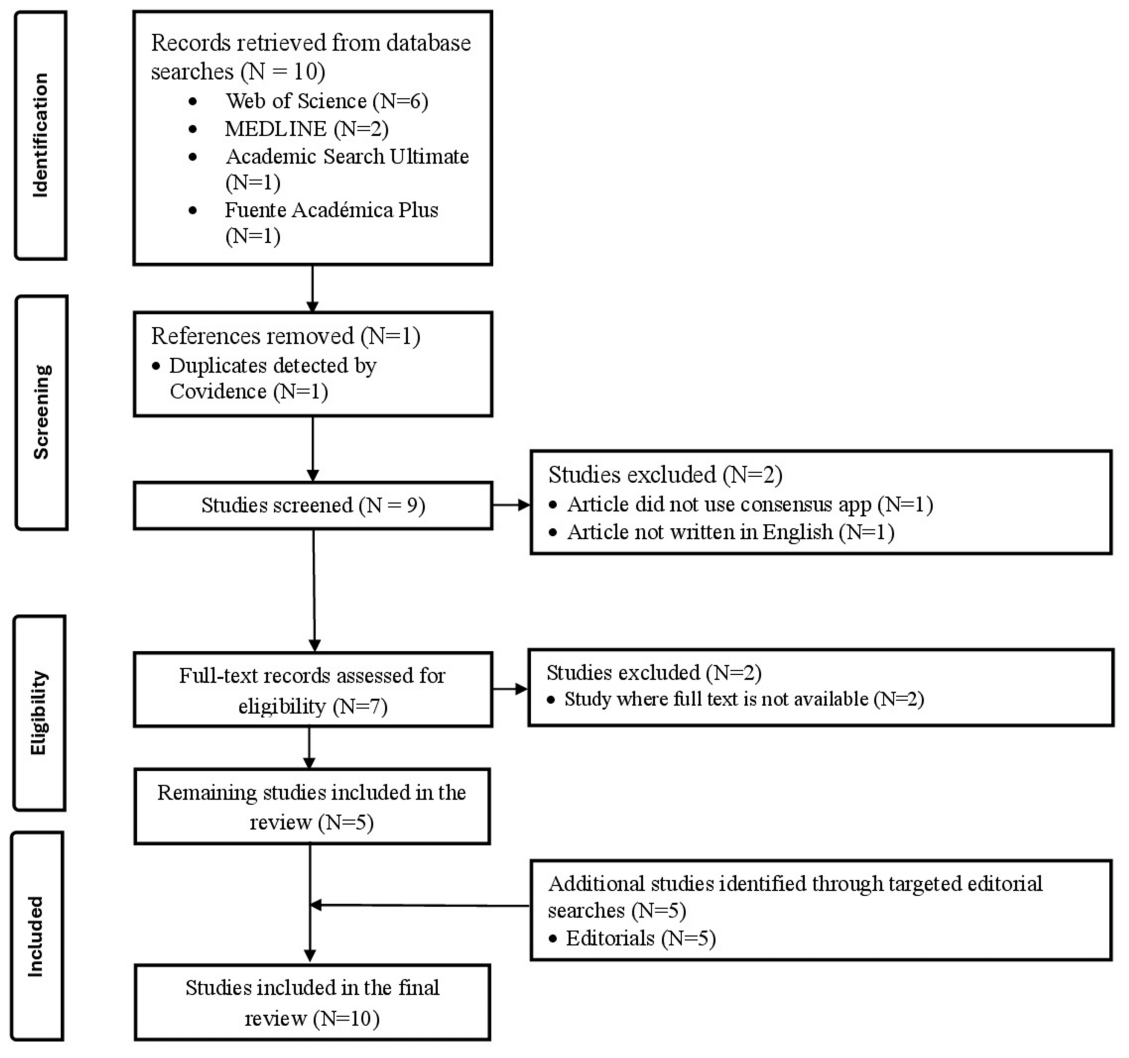
PRISMA flow diagram of articles PRISMA, Preferred Reporting Items for Systematic Reviews and Meta-Analyses

This review is based on a rapid review of the literature to see how the Consensus App is used and reported in the literature. Our review (Figure 1) followed the Preferred Reporting Items for Systematic Reviews and Meta-Analyses (PRISMA) guidelines to ensure transparency [3]. We focused on identifying applications, benefits, and ethical concerns related to the Consensus App while looking at its underrepresentation in scholarly publications.

Search Strategy

The search was conducted on December 23, 2024, across 210 academic databases available through the Texas A&M University library. We used the search terms: "Consensus App" and "Consensus AI" to identify relevant studies. Because the app was released in 2022, this review included articles published between 2022 and 2024. Of the 210 databases searched, only four returned relevant results for this study: Web of Science, Academic Search Ultimate, MEDLINE, and Fuente Académica Plus. In addition to the structured database search, we conducted a targeted editorial search to identify recent perspectives on the ethical and transparency dimensions of AI use in academic research. Five editorials were identified through manual searches in journals such as Accountability in Research, The Journal of Spinal Cord Medicine, and Journal of Cataract & Refractive Surgery, using a combination of keyword filters (e.g., “AI,” “academic publishing,” and “transparency”) and manual screening of editorial sections. These editorials were selected intentionally to seek current editorial perspectives and were later analyzed using ChatGPT-4.5 to support thematic synthesis.

Screening Process

The initial search retrieved 10 articles. One duplicate was removed using Covidence, resulting in nine articles for screening. During the screening phase, two articles were excluded: one did not reference the Consensus App, and one was not written in English. Of the seven full-text articles assessed for eligibility, two were excluded due to unavailable full texts. The remaining five studies met all inclusion criteria. In addition, five editorials were identified through targeted searches and included in the analysis. Altogether, 10 studies were included in the final review. These papers were combined in order to assess the app's potential and difficulties as well as to comprehend how it was incorporated into research.

Inclusion and Exclusion Criteria

Studies were eligible for inclusion if they were peer-reviewed journal articles or peer-reviewed conference proceedings published between 2022 and 2024, written in English, and explicitly discussed the use of the Consensus App in academic research. Editorials published during the same period were also included if they addressed ethical, transparency, or reporting practices related to the use of AI in academic work. Studies were excluded if they did not mention the Consensus App, were not peer-reviewed, were not published in English, or lacked full-text access.

Data Extraction and Thematic Synthesis

To ensure the reliability of this review, a structured data extraction framework was used to extract data systematically from each article that met the inclusion criteria. The review focused on how the Consensus App was used for literature identification, evidence synthesis, and disclosure practices. We documented the perceived benefits and ethical concerns such as citation practices and transparency of the Consensus App. In addition, we used ChatGPT-4.5 to support the thematic synthesis of five editorials discussing AI use in academic research. Specifically, the tool was used to generate initial summaries, identify recurring themes across the editorials, and help compare positions on transparency, ethical disclosure, and responsible AI integration. The outputs generated by ChatGPT-4.5 were manually reviewed, verified against original texts, and revised for accuracy and clarity before being integrated into the manuscript.

Results

Overview of Included Studies

The summary of the included studies is presented in Table 1. Five published studies between 2023 and 2024 met the inclusion criteria. All the studies used the Consensus app (sometimes referred to as “Consensus AI”) to aid in literature searches and/or content analysis alongside traditional databases (e.g., PubMed and Google Scholar). Four studies [4-7] were journal publications focusing on pediatric dentistry, socioeconomic factors in pediatric oral health, and global development indicators, respectively, while study Čep et al. [8] discussed an academic keyword search comparison.

**Table 1:** General description of the studies SES, socioeconomic status; HDI, Human Development Index; AI, artificial intelligence; UNDP, United Nations Development Program

Reference	Country	Sample/Population	Research Design	Journal	Purpose of the Study	Ethical Concerns Raised	AI Limitations Noted
[4]	Saudi Arabia	Children aged 0-12 years require pediatric dental crowns.	Narrative review of articles from PubMed, Google Scholar, and Chat.Consensus.App (last two decades).	Cureus	To examine advancements in dental crown materials for pediatric dentistry.	None stated.	None stated.
[5]	Saudi Arabia	Pediatric populations globally, with a focus on lower SES communities in both urban and rural areas. 40 peer-reviewed articles were synthesized.	Narrative review using databases (PubMed, Google Scholar, and Consensus app). The study synthesized findings from peer-reviewed articles published by Dec 2023.	Cureus	To examine the impact of SES on pediatric oral health. The focus is on disparities in dental caries prevalence and oral health-related quality.	None stated.	None stated.
[6]	Philippines	Nations (69) ranked in the UNDP's HDI.	Narrative literature review using AI tools (Consensus App, Google Bard, and ChatGPT) and validated sources like Google Scholar, Scopus, and Clarivate Analytics.	JPAIR Multidisciplinary Research Journal	The article explores financial indicators as predictors of the HDI and analyzes relationships between financial metrics and global HDI rankings.	Data privacy, impartiality, respect for cultural diversity; no specific discussion of AI ethics.	AI tools used for literature search; no reflection on AI limitations, risk of incomplete or biased results.
[7]	India	Studies and tools relevant to AI-assisted medical manuscript composition.	Narrative review using PubMed, Google Scholar, and Science Direct databases.	Cureus	The article examines the applications, benefits, limitations, and ethical considerations of AI in medical manuscript writing, offering recommendations for effective use.	Bias, misinformation propagation, data privacy, authorship issues, lack of transparency, academic integrity, and job displacement.	Potential for misinformation, lack of originality, incorrect or missing citations, overreliance on AI, limited access to subscription content, hallucinations, and reduced creativity.
[8]	Croatia	Academic professionals and higher education institutions utilizing AI tools for literature reviews and academic writing.	The study is a narrative review that employs Consensus AI and Google Scholar for literature searches.	Intelligent Computing: Proceedings of the 2024 Computing Conference, Volume 3 (Lecture Notes in Networks and Systems, Volume 1018)	The article explores the applications, benefits, limitations, and ethical considerations of AI tools like ChatGPT in higher education.	Data privacy, authorship and copyright issues, transparency, bias, ethical codex, and false data risks.	AI hallucination, surface-level analysis, exclusion of non-English/unpublished works, and reliance on human supervision.

Findings

The reviewed articles revealed that the use of the Consensus App in published academic work remains relatively low. Of the 210 databases searched, only five articles explicitly referenced their application. This may reflect either limited adoption among researchers or underreporting of its use in scholarly publications. These studies provided limited insights into how the app has been used in the literature. According to the findings, the Consensus App allows easy access to academic articles [4]. It can “identify research gaps, thereby enriching the research process” [7], but usage of the app is still limited, and disclosure of its use in published research is not yet common. This raises important ethical considerations. For instance, should researchers be required to disclose their use of AI tools like the Consensus App when such tools inform literature reviews or academic outputs?

The reviewed studies indicate that the Consensus App has primarily been used as a tool for synthesizing relevant literature. The app helped researchers in our included studies to retrieve peer-reviewed papers and summarize important findings to facilitate their work [4]. Čep et al. [8] compared traditional database searching with AI-driven methods, noting that “Consensus AI was used to quickly search for research according to keywords.” The app was mostly used in medical research alongside other AI tools like ChatGPT to improve research [4,5,7], while others relied on it as the only tool for their literature searches. However, none of the studies detailed how the Consensus app was used and which specific functions of the Consensus app were adopted for the research work compared to traditional database searches like Web of Science, Education Source Ultimate, and CINAHL Ultimate. These benefits suggest that the Consensus App could play an important role in supporting academic research if its use becomes more widely recognized.

Despite its advantages, we identified an ethical concern in the reviewed studies. A major issue is the potential oversimplification of research findings because the AI-generated summaries may not fully represent the depth of academic arguments.

Underreporting of the Consensus App in the Literature

One of the most significant findings of our review is the clear underreporting of the Consensus App in the literature. Although AI tools like ChatGPT have received widespread attention and critique, there is a paucity of data on the use of the Consensus App from published articles on AI-driven research. Several factors may contribute to this lack of reporting.

First, a lack of awareness among researchers could be another reason why it is not being reported in the literature. The Consensus App is a new platform; many researchers are probably not familiar with its features. Moreover, the hesitation of authors to disclose AI use in research may also be a factor because some researchers might avoid mentioning AI-assisted methods due to concerns about the credibility of their work [9]. The findings suggest that increasing awareness of the Consensus App and encouraging researchers to disclose their use of AI tools in publications will help bridge this gap.

Discussion

Summary of Findings

Based on the small and heterogeneous set of five peer-reviewed studies that met our inclusion criteria, we found that empirical evidence on the Consensus App remains sparse and largely descriptive. The research articles used the tool chiefly to locate, filter, and organize peer-reviewed literature; none benchmarked its recall, precision, or search speed against traditional databases or rival AI platforms. These studies highlight a pronounced gap between early, anecdotal enthusiasm for the Consensus App and the lack of rigorous, discipline-specific evaluations. Consequently, claims of superior efficiency by the developers of the app remain provisional and will require larger comparative trials and clearer disclosure practices before firm conclusions can be drawn.

Comparison With Other AI-Assisted Platforms

Compared with other AI-assisted literature platforms such as Elicit, Iris.ai, and ResearchRabbit, Scite AI, and Scopus AI, Consensus AI is distinctive in drawing exclusively from peer-reviewed sources and attaching an evidence-agreement score to each claim [1]. Elicit and Iris.ai prioritize speed and breadth by indexing pre-prints and other grey literature [10,11], whereas ResearchRabbit emphasizes citation-graph exploration without evidence grading [12]. An early head-to-head test conducted by librarians at the Hong Kong University of Science and Technology (HKUST) found that, for the query “Does sleeping less lead to weight gain?” Consensus and Scite produced the longest syntheses (approximately 10 cited studies), Scopus AI retrieved five to eight, and Elicit returned four; the librarians concluded that tools geared toward citation context or interactive matrices deliver deeper but slower analysis, while rapid-overview systems trade detail for speed [13]. These preliminary data underscore a trade-off between usability and search depth that future benchmark studies should quantify.

Limited Disclosure of AI Tool Use

The Consensus App is helpful in simplifying literature reviews by providing quick access to peer-reviewed articles and giving a summary of the findings [7]. However, the results indicate that its role in research is not well-documented in published studies. This lack of reporting raises important questions about how generally the Consensus app and other AI tools are being used and whether researchers may be reluctant to disclose the use of AI tools in their work [14,15]. One explanation for this underreporting is probably due to the app’s 2022 launch, meaning many researchers may still be unaware of its features and full capabilities. Additionally, AI-powered research tools remain a subject of debate in academic integrity discussions. Some researchers may not want to disclose their use of the Consensus App because AI-generated summaries are sometimes viewed with skepticism by journal editors and peer reviewers [16,17]. Concerns include the potential for misrepresentation of original findings, lack of critical engagement, or overreliance on AI in place of independent analysis, which can raise questions about academic integrity and authorship responsibility [18,19]. Editors are not necessarily discouraging the use of AI altogether, but they are increasingly emphasizing transparency, requiring authors to clearly state when and how AI tools were used during manuscript preparation. This aligns with the discussions on the integration of generative AI in research, as transparency in methodology is a key issue [20,21].

Balancing the Benefits and Ethical Concerns

In our review, we evaluated how the Consensus App is currently being used in academic research, with particular attention to its usability, integration into scholarly work, and the ethical implications of relying on its AI-generated summaries without fact-checking if the content generated is actually true. We found that the Consensus App makes the process of literature search faster but also highlighted the need to use it responsibly. A major concern we identified with Consensus App AI-generated summaries is the oversimplification of the research findings. While the app can extract important points from multiple studies, it may not fully reflect the arguments raised by the authors of those studies. Therefore, instead of relying solely on synthesized output from the Consensus App, researchers should take their time to read and comprehend original articles.

Citation and academic integrity present another important concern. Some researchers may intentionally fail to properly cite original sources when relying on Consensus app summaries and literature searches. This raises questions about how AI-generated research should be integrated into academic work in a way that is similar to the existing way of citing academic articles.

As Gatrell et al. [19] emphasized, transparency is critical when integrating AI tools like Consensus App into scholarly work. Authors are encouraged to clearly report how AI was used, whether for drafting, summarizing, or formatting, and to take full responsibility for the content generated. This aligns with broader calls across academic publishing to preserve authorship integrity while embracing the benefits of AI assistance [22-24].

Editorial Insights and Recommendations for Transparent and Ethical AI Use in Research

The Consensus App is a powerful literature search tool that can complement the traditional search of databases and can reduce substantial time and resources (e.g., subscribing to different databases) for researchers. Although the app can make research work more efficient, it is essential to verify the summaries generated by the AI tool while checking them with the original articles to ensure accuracy. In addition, we encourage researchers to disclose the use of AI (if any) in their work for transparency and accountability. For instance, Cheong BC [25] emphasizes that transparency enables individuals to understand how AI systems influence decision-making, while accountability ensures that there are mechanisms for assigning responsibility and providing redress when AI causes harm. He argues that implementing transparency is essential not only for ethical AI governance but also for safeguarding societal well-being​. Similarly, El Ali et al. [26] advocate for disclosure practices grounded in Article 52 of the European Union’s AI Act [27]. They call for transparent identification of AI-generated content and define disclosure as “making known or public” the nature of AI involvement. Their study contributes a framework of 149 questions to guide future research on responsible AI disclosure across human-computer interaction contexts​.

To promote transparency and integrity, we propose a set of minimum disclosure standards for researchers using AI tools, such as the Consensus App. These standards include stating the version of the AI tool used (e.g., Consensus 2.0, ChatGPT-4.5, and Gemini 1.2), describing how the tool was applied (e.g., literature searching, summarizing articles, and drafting text), listing the databases accessed through the AI tool, outlining any preliminary findings generated prior to verification, and explaining the validation process (e.g., comparing to original articles or cross-checking references) before incorporating the AI-generated output into the final manuscript.

These practices will help ensure transparency, reproducibility, and academic integrity when using AI tools in research. Adopting these standards will enable editors, peer reviewers, and readers to assess the use of AI tools more clearly, helping to ensure that their integration into academic research is guided by accountability and transparency.

To expand the above recommendations, we used ChatGPT-4.5 to synthesize and compare the main themes from five editorials [19,28-31] that discuss the usage of AI tools in scholarly work. These editorials emphasized the need for transparent reporting practices and offered both general guidance and specific insights on effectively integrating AI into research.

One common theme was the importance of transparency in AI use. All five editorials emphasized that authors must disclose the use of AI tools in the research process. This includes specifying whether AI assisted with literature reviews, data extraction, drafting sections of the manuscript, or editing. Transparent disclosure enables readers, reviewers, and editors to assess the contribution of AI to scholarly work accurately.

Another central theme was the accountability of the authors. Although AI tools can streamline certain tasks, the editorials stated that authors bear ultimate responsibility for the content they produce. Researchers must scrutinize and evaluate AI-generated outputs for accuracy and ensure they uphold scholarly and ethical standards.

The editorials also highlighted the limitations of AI tools. Even sophisticated language models may misinterpret context, overlook important points, or introduce bias. Authors are advised to compare AI-generated content with original sources and reference materials to validate its accuracy and completeness.

Finally, ethical concerns and academic integrity were shared concerns across the editorials. The risk of plagiarism, improper attribution, and blurred lines of authorship are heightened when AI tools are involved. The editorials advocate for the development of clear guidelines that define acceptable and unacceptable uses of AI, particularly in drafting content or processing unpublished data. These steps are essential to maintaining the credibility and integrity of academic publishing.

Authorship and Plagiarism

Shifting attention to authorship and plagiarism, Dupps [29] raised concerns regarding authorship attribution when writing with AI. Specifically, the editorial highlighted the challenge of identifying subtle forms of AI-assisted plagiarism, which may not be detected by conventional detection software. He emphasized the need for authors to clearly distinguish between their intellectual contributions and those written by AI. As AI writing becomes more prominent, so too must the rigor with which researchers acknowledge and contextualize their use of such tools. Gatrell et al. [19] expand the discussion by introducing specific guidelines for editors and reviewers, prohibiting the use of AI-based tools in peer review to maintain confidentiality and safeguard the integrity of the evaluation process. Their policy framework, which details the AI applications that are permissible and those that are not, reflects the increasing institutional demand for clear and transparent reporting of AI in academic writing.

Murphy et al. [30] underscore the importance of responsible AI use by calling on authors to fully disclose their use of generative AI during manuscript preparation. Their editorial cautions against AI-assisted plagiarism, particularly subtle forms that may escape traditional detection methods. In response to growing concerns about “fake science,” they recommend editorial safeguards such as enhanced plagiarism screening, author verification, and clearer authorship policies to maintain academic integrity. Their guidelines stress that AI tools like ChatGPT should not be listed as authors and that human researchers must take full responsibility for content accuracy, originality, and ethical compliance. This view aligns with Hosseini et al. [31], who stress that authors remain fully responsible for any factual inaccuracies or ethical breaches introduced by AI-driven systems. By explicitly distinguishing human from AI-generated intellectual work, researchers uphold accountability and reinforce the clarity and trustworthiness of academic publishing.

Limitations and Future Directions

While this review offers important insights into the Consensus App’s potential and ethical implications, several limitations must be acknowledged. Although we searched 210 academic databases, only five peer-reviewed studies met the inclusion criteria. This discrepancy highlights a potential disconnect between the growing use of AI tools in academic work and their formal documentation in scholarly literature. Possible explanations include the relative novelty of the Consensus App, underreporting by authors, or limited discoverability due to indexing and platform visibility. Moreover, the review was limited to English-language publications, which may have introduced language bias and excluded studies from regions with emerging AI research. The publication window (2022-2024) also coincides with the early adoption phase of the app, potentially omitting relevant ongoing or unpublished work.

Furthermore, drawing on the HKUST library’s head-to-head comparison of AI literature tools [13], we qualitatively contrasted Consensus with Elicit, Iris.ai, ResearchRabbit, Scite AI, and Scopus AI, but we did not apply a formal evaluation framework; future studies should therefore adopt clear, quantitative benchmarks, such as retrieval accuracy, search speed, interface usability, transparency, and reproducibility to enable rigorous, replicable head-to-head assessments.

Beyond Consensus, several AI-assisted literature platforms, Elicit, Iris.ai, ResearchRabbit, Litmaps, and Semantic Scholar’s TLDR function, also use concept-level retrieval and rapid summarization. They were outside the scope of the present review because they have not yet been examined in peer-reviewed studies, but future work should benchmark them alongside the Consensus app and conventional databases to establish their relative strengths and limitations.

In addition, the scope of this review was limited to English-language publications, which reinforces the potential for language bias. Regional adoption and institutional preferences for certain AI tools may also influence which studies are documented and indexed. Future research should investigate how the use of the Consensus App varies across global contexts and academic disciplines, and whether cultural or policy-related factors influence the reporting of AI-supported research.

Beyond technical performance, future studies should also examine how diverse AI tools are being used and disclosed in academic work. Researchers should propose standardized guidelines for transparent reporting, including which specific Consensus App features (e.g., Consensus Meter and Pro Analysis) were used in the literature search. Although these features were not explicitly described in the reviewed studies, they may play a significant role in research workflows and outcomes. Encouraging detailed tool reporting will help readers better understand the practical application of AI tools in scholarship.

Finally, future studies should prioritize mixed methods approaches and incorporate author interviews to offer deeper insights into AI adoption and disclosure practices. This would contribute to a more balanced and context-sensitive understanding of how AI is shaping academic writing, research transparency, and scholarly integrity.

## Conclusions

This review explored how the Consensus App is being used in academic research and found that it can make research easier by helping scholars find and organize studies more efficiently. While the Consensus App may enhance research efficiency and support evidence-based inquiry, uptake in peer-reviewed publications remains limited. Researchers have raised concerns about the accuracy, transparency, and ethical challenges of using generative AI tools in scholarly work, particularly regarding authorship attribution, bias, and reproducibility. Further empirical investigation is needed to understand the barriers to integrating tools like Consensus into academic workflows. Validated, evidence-graded syntheses produced by Consensus could also inform evidence-based decisions in higher-education policy and curriculum design, extending the app’s relevance beyond day-to-day literature search.

As AI tools continue to influence how research is conducted, they are raising important questions about fairness, transparency, and responsibility. Using AI without clear rules can lead to confusion about authorship or hidden contributions, which can affect the credibility of academic work. To prevent this, universities, journals, and researchers must work together to develop clear guidelines for how AI should be used and acknowledged. At the researcher level, scholars can mitigate these risks by disclosing the app version, search tier, and date in their Methods sections and by checking AI-generated summaries against the full texts they cite, thereby minimizing oversimplification and citation drift. When applied with care and openness, tools like the Consensus App have the potential to improve research quality while maintaining the values of honesty, reliability, and academic integrity.
